# Kinetics of Holographic Recording and Spontaneous Erasure Processes in Light-Sensitive Liquid Crystal Elastomers

**DOI:** 10.3390/ma5050741

**Published:** 2012-04-25

**Authors:** Marko Gregorc, Hui Li, Valentina Domenici, Gabriela Ambrožič, Martin Čopič, Irena Drevenšek-Olenik

**Affiliations:** 1 J. Stefan Institute, Jamova 39, Ljubljana, SI 1000, Slovenia; E-Mails: mark.gregorc@gmail.com (M.G.); martin.copic@fmf.uni-lj.si (M.C.); 2 Nankai University, TEDA APS, 23 Hongda Street, Tianjin 300457, China; E-Mail: turtle_007@126.com; 3 Dipartimento di Chimica e Chimica Industriale, Università degli studi di Pisa, via Risorgimento, 35, Pisa 56126, Italy; E-Mail: valentin@dcci.unipi.it; 4 National Institute of Chemistry, Hajdrihova 19, Ljubljana, SI 1000, Slovenia; E-Mail: gabriela.ambrozic@ki.si; 5 Center of Excellence for Polymer Materials and Technologies, Tehnološki Park 24, Ljubljana, SI 1000, Slovenia; 6 Faculty of Mathematics and Physics, University of Ljubljana, Jadranska 19, Ljubljana, SI 1000, Slovenia

**Keywords:** liquid crystal elastomers, light-sensitive materials, holographic lithography, optical microstructuring, recording kinetics

## Abstract

The optical mechanism for imprinting one-dimensional grating structures into thin films of a light-sensitive monodomain liquid crystal elastomer is investigated by analyzing the time dependence of optical diffraction properties. The recording kinetics shows an irregular oscillatory behavior, which is most expressed at small grating spacings and at temperatures close to the nematic-isotropic phase transition. The oscillations are attributed to the opto-mechanical response of the film, *i.e*., to contraction of the film during the recording process. At temperatures far below the nematic-isotropic phase transition, the spontaneous erasure kinetics exhibits exponential relaxation with relaxation time following the Arrhenius activation law. However, at temperatures close to the nematic-isotropic phase transition, the erasure process shows an interesting nonmonotonic behavior that we attribute to the non-linear relation between the concentration of the photo-transformed chemical groups and the nematic order parameter.

## 1. Introduction

Liquid crystal elastomers (LCEs) are cross-linked polymer materials that exhibit strong coupling between the conformational state of the polymer chains and orientational order of the mesogenic (liquid crystalline (LC)) molecular units. They display several interesting mechanical properties, such as large spontaneous shape modifications during cooling and heating or unusual soft elasticity during stretching [1]. Light-sensitive liquid crystal elastomers (LS-LCEs), in addition, also exhibit a coupling between the LC orientational order of the mesogenic units and the conformational state of the photosensitive molecular units, for instance of photoisomerizable azobenzene derivatives [2,3,4,5,6,7]. This coupling causes a cooperative response, due to which even relatively minor photo induced perturbations in the concentration of *trans*- and *cis*-isomers cause large modifications of the LC order parameter and consequently of the refractive index of the medium [8,9,10]. This provides a possibility to record volume phase holograms of high efficiency. Due to the rubber elasticity of the polymer network, the recorded holographic structures can be reversibly expanded and contracted, which is very interesting for applications in mechanically tunable diffractive optical devices [11].

The recording capability is additionally improved by using so-called monodomain LCEs, known also as liquid single crystal elastomers [12]. These are specifically prepared LCE films, which exhibit a spatially homogeneous (aligned) orientation of the mesogenic molecular units on a macroscopic scale. Holographic patterning in such films results in modifications of the optical birefringence of the medium [13]. Due to optical birefringence recording and reading properties are sensitive to the polarization state of the optical beams [14], which gives rise to some additional interesting features, such as a possibility to fabricate polarization holographic gratings [15].

The dynamic response of LS-LCEs to optical irradiation has been studied predominantly in relation with their opto-mechanical properties. Either light-induced modifications of the sample shape or variations of the internal stress whilst keeping the shape fixed were investigated using mainly spatially homogeneous ultraviolet (UV) irradiation [3,5,7,8,13,16,17]. Response times from a few seconds up to several hundreds of minutes were observed. However, in configurations in which photothermal heating dominates over the photoizomerization effect, much faster responses can be observed [18,19,20,21]. In our experiments inhomogeneous illumination with UV laser light forming a spatially periodic intensity pattern was used. It generates a periodic modification of the optical absorption and refractive index of the sample, *i.e.*, it leads to the formation of an optical diffraction grating. The diffractive properties of the grating were investigated by a probe laser beam with a wavelength in the visible spectral range. The particularity of our method is that the signal originates predominantly from the surface region of the sample in which the UV absorption and the associated *trans-cis* isomerization take place [10], whereas the opto-mechnical response probes modifications of the entire volume of the sample.

## 2. Experimental Section

**Figure 1:**
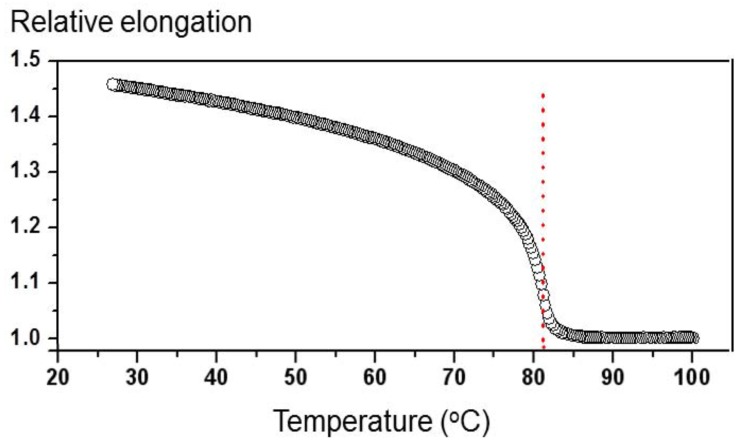
Relative spontaneous elongation of the sample as a function of temperature measured in the absence of UV illumination. The dotted line indicates the temperature maximum of (*dL*/*dT*), which is considered as the transition temperature from the nematic to the paranematic phase.

Monodomain side-chain LCE films with a thickness of 150 μm were prepared according to the two-step “Finkelmann crosslinking procedure” [12]. The polymer backbone is based on a commercial hydroxymethyl-polysiloxane, which is cross linked by 1,4-bis (undec-10-en-1-yloxy) benzene used as cross-linker unit. The side-chain moieties are composed of usual rod-like mesogens (4-methoxyphenyl 4-(but-3-en-1-yloxy) benzoate) and light-sensitive azomesogens (1-(4-(hex-5-enyloxy)phenyl)-2-(4-methoxyphenyl) diazene) in the ratio of 9:1. Details of the sample fabrication and characterization procedures are described elsewhere [22]. In the absence of the UV illumination, the nematic-isotropic (more accurately nematic-paranematic) phase transition occurs at *T*_c0_ = 82 °C, where index 0 denotes zero concentration of the *cis* isomers. The dependence of the associated relative spontaneous elongation of the sample in the direction of the nematic director as a function of temperature is shown in Figure 1.

**Figure 2:**
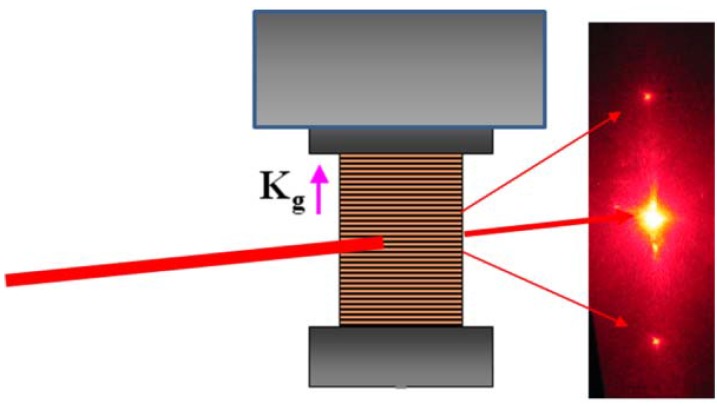
Schematic drawing of the experimental configuration: The sample (striped rectangle) is fixed at its upper edge while its lower edge is loaded with a small weight. On the right: Image of the far-field diffraction pattern of the probe beam. The ±1st order diffraction peaks are analyzed in the experiments.

Optical experiments were performed with films of the size of 5 mm × 5 mm. The upper edge of the film was fixed to the frame on the heating stage, while the lower edge was free to move. However, to prevent bending of the film, the lower edge was loaded with the small weight of a mass of 0.5 g (see Figure 2). Diffraction gratings were recorded with two intersecting UV laser beams from an argon ion laser operating at a wavelength of *λ_R_* = 351 nm. The power density of each beam was 12.7 mW/cm^2^. The intersection angle between the beams was varied in the range from 2° to 20°, so that the grating spacing *Λ* of the resulting 1D transmission gratings was in the range 1 µm < *Λ <*10 µm. The grating vector **K_g_** = (2*π*/*Λ*)**e_g_** was parallel to the nematic director **n**. The polarization of the writing beams was also parallel to **n**, *i.e.*, the beams were extraordinarily polarized. The diffraction efficiency of the gratings was probed with a low-power beam from a HeNe laser operating at a wavelength of *λ_P_* = 632.8 nm. The probe beam entered the film at normal incidence and was also extraordinarily polarized. The diameter of the probe beam was about 0.2 mm. The intensities of the 1st order diffraction peaks were measured with photodiodes (Figure 2). Typical UV light-induced modifications of refractive index in azo-doped LCEs are in the range *n_1_*~10^−2^ [7,8], while the absorption depth *d* is only a few micrometers [10]. Therefore a relatively weak diffraction is expected with a diffraction efficiency *η* = *I*_±1_/*I*_p_ (where *I*_±1_ and *I*_p_ denote the intensities of the ±1st order diffraction peaks and of the incident probe beam, respectively) in the range *η ~*(*πdn_1_/λ_P_*)^2^~0.05 [23]. The diffraction regime at the boundary between the Raman-Nath and the intermediate regime is envisaged [23].

## 3. Results and Discussion 

**Figure 3:**
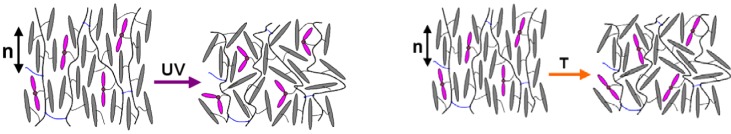
Schematic illustration of the effect of UV-light-induced *trans-*to-*cis* isomerization (left) and heating (right) on the orientational order of a liquid crystal elastomers (LCE). Pink colored double ellipsoids denote azomesogenic side groups and gray colored ellipsoids denote usual mesogenic side groups. Black lines denote polymer chains and blue lines denote cross-links between them. Both isomerization and heating produce a decrease of the LC order parameter *S.* Consequently, contraction of the film in the direction of the nematic director **n** and elongation in the direction perpendicular to **n** take place.

Typical measured values of the diffraction efficiency *η* at temperatures 25 °C < *T* < 82 °C were in the range of 1%–5%. For *T* > 82 °C, the diffraction efficiency was drastically decreasing with increasing temperature and at *T* = 90 °C its value was more than two orders of magnitude smaller than at *T* < 82 °C. This observation signifies that the presence of LC orientational order is crucial for efficient holographic recording. The origin of the recording process induced by UV light at all temperatures is the same, namely *trans*-to-*cis* isomerization of the azomesogene (diazene) side groups. However, at *T* < 82 °C the conformational state of these side groups is strongly coupled with the orientational order of the non-photosensitive mesogenic side groups, which means that an increased concentration of the *cis* groups *N*_c_ causes a decrease of the scalar nematic order parameter *S* (*S* =< (3cos^2^*θ* − 1)/2> (where *θ* is the angle between the long axis of the mesogenic molecules and **n**). The decrease of *S* results in spontaneous contraction of the sample in the direction of **n**, very similar to the effect of the increasing temperature [12]. The outcome of both perturbation mechanisms (*trans-*to-*cis* isomerization and heating) is schematically illustrated in Figure 3. Important for holographic recording is that a decrease of *S* also results in the proportional decrease of the optical birefringence of the material *Δn* = *Δ*(*n*_e_−*n*_o_) ∝ *ΔS* (where *e* and *o* denote extraordinary and ordinary ray, respectively), which can have values as large as Δ*n*~0.1 [8,9]. For *T* > 82 °C, the nematic orientational order is drastically reduced and its coupling with the conformational state of the azomesogenic side groups practically vanishes. Thus modifications of the refractive index can take place only via direct modification of the optical polarizability of azo-molecules, *i.e.*, via modification of the contribution of the azo molecules to the net refractive index of the material at the probe wavelength *λ_P_*, and consequently the resultant patterning effect is much weaker. At high temperatures azo-doped LCEs become similar to the conventional azo-doped elastomeric materials.

For a sinusoidal interference pattern of the UV light intensity with *I = I*_0_(1 + *Cos***K_g_r**), the spatial dependence of the concentration of *cis* isomers *N_c_* can be written as [2,3]:

(1) $$N_{c}(r,t)=\frac{N_{0}A\tau \;(1+CosK_{g}r)}{1+A\tau (1+CosK_{g}r)}\left[1-e^{-t/\tau_{eff}}\right]$$

where **r** is a coordinate vector, *t* is the recording time, 1/*τ_eff_* = *A*(1 + *Cos***K_g_r**) + 1/*τ*, *A* is a rate constant of the UV-light-induced *trans*-to-*cis* isomerization, which is proportional to *I*_0_, *τ* is the relaxation time of the thermally-induced *cis*‑to-*trans* back-isomerization, and *N_o_* is the total concentration of the azomesogens. At this point we need to mention that Equation 1 is valid only for the top-most layer of the sample. Due to the strong absorption of the UV light, the intensity of recording radiation decreases with increasing sample depth and consequently the recording process is less effective at larger depths. Our recent investigation of this problem using numerical simulations revealed that in the beginning of the UV illumination the grating structure is recorded in a surface region with an effective thickness of 2 μm [10]. However, at longer illumination times a reduced concentration of the absorptive *trans*-azomesogens in the surface layer causes an increase of the penetration depth of the UV light and subsequently at very long illumination times the effective thickness of the grating structure increases to 20 μm. From this one can anticipate that, although the kinetics of the *trans*-*cis* isomerization process itself is in general quite simple, its dependence on spatial variations of the UV light intensity and its non-local coupling to the LC orienational order will in total bring a complicated time dependence of the refractive index modifications during holographic recording. In contrast, the relaxation (spontaneous erasure) of the grating structure is governed only by the thermally-induced *trans*-to-*cis* back-isomerization and should therefore behave much more simply. The relaxation of the concentration of cis-isomers *N*_c_ can be written as [2,3]:

(2) $$N_{c}(r,t)=N_{c}(r,0)e^{-t/\tau }$$

where *N*_c_(**r**, 0) denotes the profile established at the end of the recording process.

The main difference between Equations 1 and 2 is that the effective recording time *τ_eff_* in Equation 1 strongly varies with position in the sample, as it depends on the intensity of the UV light that is changing along the direction of the grating vector **K_g_** as well as with the depth of the sample. The relaxation time *τ* in Equation 2, on the other hand, is supposed to be more or less spatially independent, because the thermal gradients in the sample are suppressed by heat diffusion.

**Figure 4:**
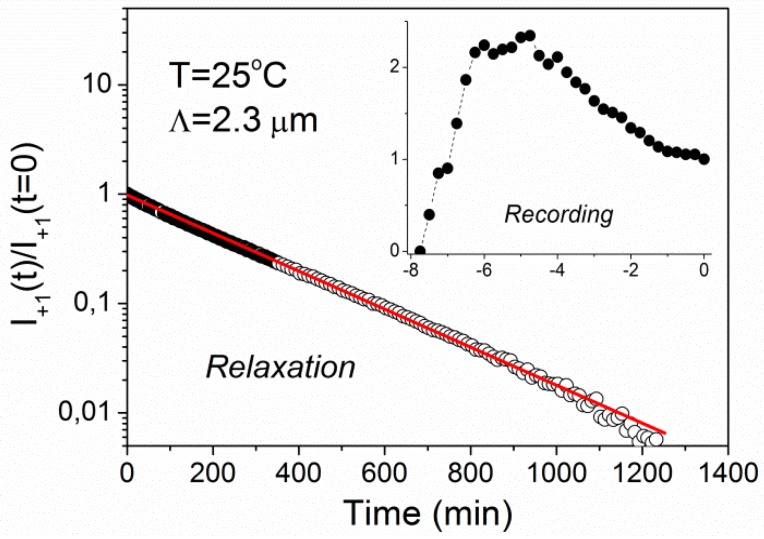
Time dependence of diffracted intensity during recording (inset) and relaxation for a grating with the period of Λ = 2.3 μm at *T* = 25 °C. The solid line is a fit to single-exponential decay.

Let us now shift the discussion from the expectations to the experimental results. Figure 4 shows time dependence of the intensity of the +1st order diffraction peak measured during recording of a holographic grating with the grating period of 2.3 μm at *T* = 25 °C and its subsequent relaxation. The value *t* = 0 corresponds to the end of recording process (*i.e.*, beginning of relaxation process). The intensity is normalized to *I*_+1_ = 1 at *t* = 0. The relaxation kinetics exhibits a simple single-exponential decay with relaxation time *τ* = 247 ± 1 min. The recording kinetics (see inset of Figure 4) shows a nonmonotonic behavior with a maximum at the recording time of about 3 min.

We assume that at *T* = 25 °C the profile of the extraordinary refractive index modulation Δ*n_e_*(**r**) follows the profile of *N*_c_(**r**), *i.e.*, that we have Δ*n_e_*(**r**)  ∝ΔS(r)∝ *N*_c_(**r**). Then the nonmonotonic behavior during recording can be attributed to a saturation of the concentration of *cis*-isomers. Taking into account that in our experiments *τ* is large compared to A^−1^, Equation 1 can be simplified so that only the exponential term in the square brackets is retained. For short recording times the dependence of *N*_c_(**r**) is then sinusoidal, *i.e.*, it can be described as

(3) $$N_{c}(r)\approx N_{0}t/\tau_{eff}\approx N_{0}At(1+CosK_{g}r)={\bar{N}}_{c}(1+CosK_{g}r)$$

while for long illumination times one obtains *N*_c_(**r**) ≈ *N*_0_ everywhere except at the minima of the interference pattern, where *N*_c_(**r**) = 0. According to this, the intensity of the ±1st order diffraction peaks, which is proportional to the fundamental Fourier component of the refractive index modulation, increases in the beginning of the recording process and then decreases at long recording times. Therefore, at the same time one might start awaiting higher order diffraction peaks associated with higher harmonics to appear. As shown in a recent paper of Fally *et al.* [24] such behavior is actually expected in all kinds of two-state photo-excitable systems.

In LS-LCEs, however, there are also other phenomena that might as well lead to the result shown in the inset of Figure 1. As already mentioned before, due to photo-induced “bleaching” of the absorptive *trans* isomers, the effective thickness of the recorded holographic grating increases with increasing recording time. Consequently, the diffraction properties are shifting from the thin to the thick grating regime and the width of the Bragg peak decreases [23]. Because in our experiments the probe beam was entering the sample at normal incidence, this can bring a nonmonotonic dependence of diffraction intensity on the recording time. To further resolve this phenomenon, systematic investigations of the angular dependence of the diffraction properties for different recording times are needed, which is out of the scope of this paper. Another possibility is to use samples with the thickness much smaller than the penetration depth of the UV light.

An additional phenomenon that can cause a nonmonotonic dependence is self-diffraction of the recording UV beams. It can alter the original profile of the interference pattern, for instance by “leakage” of the intensity into higher diffraction orders. While a probe beam at *λ_P_* = 632.8 nm experiences only the refractive index modulation, the recording beam experiences a combination of both, refractive index and absorption modulation, which makes the situation more complicated [25]. This problem, which was already partially considered in the framework of paraxial approximation in [10], also requires further analysis.

**Figure 5:**
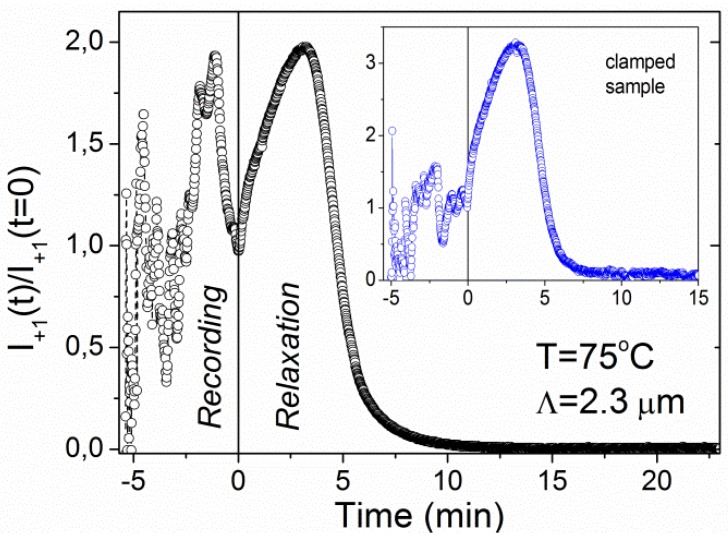
Time dependence of the diffracted intensity during recording and relaxation of the grating with *Λ* = 2.3 μm at *T* = 75 °C. The inset shows a result obtained for the clamped sample (stretched 5% with respect to the initial length).

At higher temperatures some additional features appear. Figure 5 shows recording and relaxation kinetics of a grating with *Λ* = 2.3 μm fabricated at *T* = 75 °C. The UV beams were terminated at *t* = 5.4 min, which is marked by the dashed vertical line. During the recording process diffracted intensity shows very irregular behavior of the oscillatory type. But, even more surprisingly, the relaxation kinetics becomes nonmonotonic too. In subsequent repetitions of the experiment, the features observed during the recording were qualitatively similar, but the details were different. In contrast to this, the relaxation kinetics was well reproducible.

**Figure 6:**
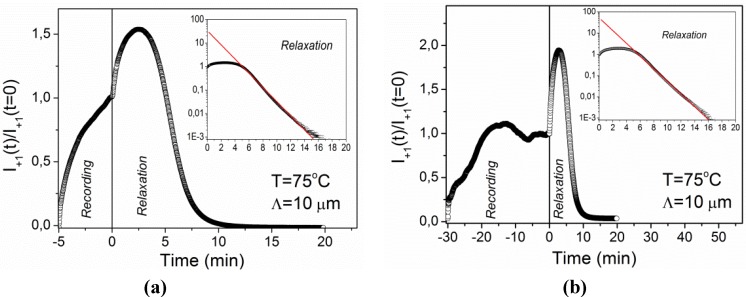
Time dependence of diffracted intensity during recording and relaxation of the grating with *Λ* = 10 μm at *T* = 75 °C: (**a**) recording for 5 min; (**b**) recording for 30 min. The insets show the relaxation process on a logarithmic scale. Solid lines are fits to single-exponential decay.

The experiment was repeated by recording a grating with a grating period of *Λ* = 10 μm under the same conditions. The result is shown in Figure 6a. In this case the recording kinetics is monotonic and quite smooth, while the relaxation kinetics exhibits the same features as for the grating with *Λ* = 2.3 μm. Additional analysis of the relaxation process reveals that at long times after the termination of the UV exposure diffracted intensity decays exponentially. The fit to exponential decay (taking into account only data for relaxation times *t*_rel_ > 5 min) is shown in the inset of Figure 6a and gives a relaxation time of *τ =* 1.47 ± 0.01 min. The same type of grating was also recorded for 30 min (Figure 6b). In this case the recording kinetics is nonmonotonic and resembles the features observed for *Λ* = 2.3 μm at *T* = 25 °C (Figure 4), while the relaxation kinetics is the same as for 5 min of recording (see inset of figure 6b).

The usual explanation for the irregular behavior of the diffracted intensity during recording is vibrations in the experimental setup. Due to the vibrations, the positions of maxima and minima in the intensity profile of the recording beams shift with respect to the recording material, which causes smearing of the holographic pattern. In LCEs, however, such dynamic shifting does not occur only due to the external vibrations, but is an intrinsic property of the material itself. This is because, as discussed in the beginning of this section, light-induced *tran*-to-*cis* isomerization causes spontaneous shrinkage of the surface layer of the sample, which in general leads to bending. In addition to this, *tran*-to-*cis* isomerization also causes heating [3], which generates shape modifications in the entire sample volume due to heat diffusion. These modifications strongly affect the diffraction efficiency, if length contraction Δ*L* of the film along the nematic director **n** is larger than *Λ*. As can be seen from Figure 1, the same modification of temperature (or *N*_c_) in the vicinity of *T*_c_ produces much larger modifications of the sample length as at room temperature. Due to this, the effect is much more pronounced at *T* = 75 °C.

One apparently straightforward solution of the problem is to clamp the film to prevent its contraction. However, our preliminary investigations showed that oscillations in clamped samples are indeed significantly reduced, but they do not completely vanish (see inset of Figure 5). We assume that this is due to the structural inhomogeneities in the film, which cause some regions of the film to contract on behalf of the stretching of the others. A similar effect was recently observed during holographic recording in clamped films of azobenzene thermoplastic elastomers [26]. One possible way to eliminate or at least reduce such problems can be to put the sample in between the glass plates.

Our results indicate that extensive systematic investigations are needed before any relevant quantitative analysis of the recording kinetics is possible. Therefore, in the following, the discussion will focus on the erasure (relaxation) kinetics. At all investigated temperatures, after an initial phase, time dependence of diffraction intensity could have been well fitted with a single-exponential decay. This decay is related to the thermally driven process of *cis*-to-*trans* back isomerization as given by Equation 2. Considering a linear relation between refractive index modification and concentration of the *cis* isomers, Δ*n_e_*(**r**) ∝ΔS(r)∝*N*_c_(**r**), and a quadratic dependence of the diffraction efficiency on refractive index modulation *η* ∝ (Δ*n_e_*)^2^ [24], that is valid at low diffraction efficiencies observed in our samples, one obtains

(4) $$I_{\pm 1}(t)=I_{p}\eta \propto e^{-2t/\tau }$$

**Figure 7:**
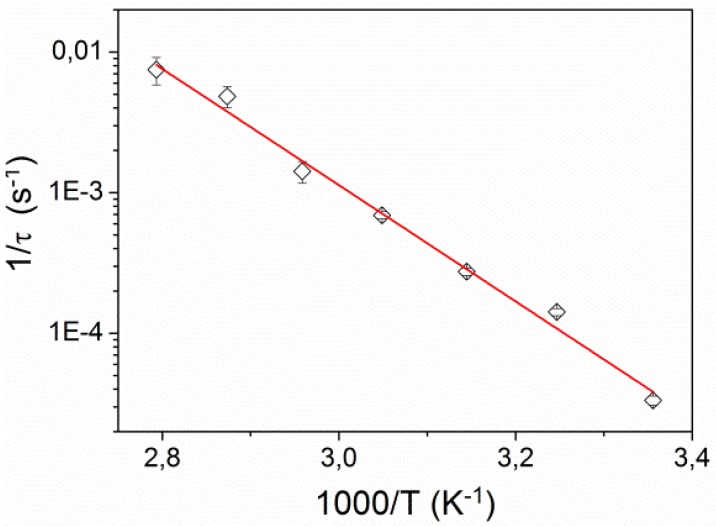
Inverse relaxation time 1/*τ* as a function of inverse temperature. The solid line is a fit to the Arrhenius relation.

Figure 7 shows the dependence of the corresponding inverse relaxation time 1/*τ* on inverse temperature. It exhibits the Arrhenius behavior

(5) $$\frac{1}{\tau }=Ce^{-E_{a}/k_{B}T}$$

where *k_B_* is the Boltzmann constant and *E*_a_ is activation energy. We found *E*_a_ = 0.87 ± 0.05 eV (83.5 ± 5 kJ/mol), which is very similar to activation energies found for *cis*-to-*trans* isomerization of azobenzene molecules incorporated into other LCE matrices and polymer hosts [16,27,28].

**Figure 8:**
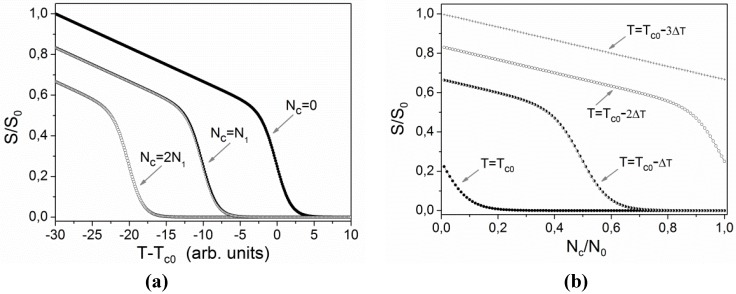
Qualitative illustration of the effects of temperature *T* and concentration *N*_c_ of *cis* isomers on the order parameter *S*: (**a**) dependence on temperature at three fixed values of concentration; (**b**) dependence on concentration at four fixed temperatures.

The nonmonotonic relaxation kinetics (Figure 5 and Figure 6) observed in the interval (*T*_c0_ − 2.0 K) < *T* < *T*_c0_, is in our opinion related to two properties: (i) the fact that our gratings are smeared and therefore have a low contrast, and (ii) the breakdown of the linear relationship ΔS(r)∝ *N*_c_(**r**) in the vicinity of *T*_c0_. The conventional theoretical model for LS-LCEs assumes that *cis* isomers act as impurities in the nematic phase and consequently cause a decrease of the transition temperature *T*_c_ [2,3,16]. When *N*_c_(**r**) = 0 (in the dark), the temperature dependence of *S* in our type of samples is proportional to the temperature dependence of the spontaneous elongation of the sample as shown in Figure 1 [22]. When *N*_c_(**r**) > 0, the dependence *S(T − T_c_)* remains the same, but the value of *T*_c_ decreases proportionally to the value of the *N*_c_, *i.e.*, *T*_c_(*N*_c_) = *T*_c0_ − *βN*_c_, where *β* is a proportionality constant [3]. The corresponding behavior is illustrated in Figure 8a using a tilted sigmoidal function *f*(*x*) = (*a* − *bx*)/(1 + e^*x*^), where *x*∝(*T − T_c_*) and *f*∝*S*, to qualitatively describe the dependence of *S*(*T*) in the vicinity of *T*_c_. Figure 8b shows the associated dependence of *S*(*N*_c_)/*S*_0_, where *S*_0_ is order parameter at some reference temperature, for four different temperatures. One can notice, that for temperatures slightly below *T*_c0_ the behavior of *S*/*S*_0_ as a function of *N*_c_/*N*_0_ is very nonlinear. In accordance to this a derivative *dS*/*dN*_c_ exhibits a pronounced maximum at some specific value of *N*_c_.

Due to smearing of the grating structure a spatial variation of *N*_c_, which would be in the ideal case given by Equation 3, is in reality much weaker and can be described as

(6) $$N_{c}(r)\approx {\bar{N}}_{c}+\Delta NCosK_{g}r$$

where Δ*N* << ${\bar{N}}_{c}$. During the relaxation process both, ${\bar{N}}_{c}$ and Δ*N*, decrease exponentially with time, *i.e.*, ${\bar{N}}_{c}(t)$ = ${\bar{N}}_{c}(0)$e^-*t*/τ^ and Δ*N*(*t*) = Δ*N*(0)e^−*t*/τ^. The associated spatial variation of the order parameter *S* can be approximately described by the first two terms of the Taylor series expansion

(7) $$S(r,t)=S\left({\bar{N}}_{c}(t)+\Delta N(t)CosK_{g}r\right)=\bar{S}(t)+\Delta S(r,t)\approx S({\bar{N}}_{c}(t))+\frac{dS}{dN_{c}}{|}_{{\bar{N}}_{c}(t)}\Delta N(t)CosK_{g}r$$

where *dS*/*dN*_c_ is evaluated at ${\bar{N}}_{c}(t)$. In Figure 8b one can see that for temperatures slightly below *T*_c0_ (see for instance the curve for *T* = *T*_c0_ − Δ*T*) the derivative *dS*/*dN*_c_ is practically zero for large values of *N*_c_/*N*_0_, then at (*N*_c_/*N*_0_)~0.5 it attains a maximum and at lower values of *N*_c_/*N*_0_ it becomes constant. This essentially means that the same relative variation of concentration of *cis* isomers $\Delta N/{\bar{N}}_{c}$ causes the largest relative variation of the order parameter Δ*S/S*_0_ at some intermediate value of ${\bar{N}}_{c}(t)$. The refractive index modulation Δ*n*_e_(**r**, *t*) is proportional to order parameter variation Δ*S(***r***,t*) [8]. From this it follows that, despite the fact that Δ*N*(*t*) in Equation 7 decays exponentially, its multiplication with *dS*/*dN*_c_, which exhibits a maximum at some specific value of ${\bar{N}}_{c}(t)$, can lead to an overall nonmonotonic dependence of the diffracted intensity during relaxation of the grating. At temperatures considerably below *T*_c0_ (see for instance the curve for *T* = *T*_c0_ − 3Δ*T* in Figure 8b) the value of *dS*/*dN*_c_ is practically constant and consequently Δ*S*(**r**, *t*) also Δ*n_e_*(**r**, *t*) decreases exponentially with time. We think that a similar scenario should work in all systems in which there exists a strong non-linear relation between the concentration of *cis* and *trans* isomers and the refractive index of the medium. However, if the transition related to the phenomenon is not continuous, the effect is much more difficult to be observed experimentally.

## 4. Conclusions 

Our results show that holographic recording and relaxation processes in LS-LCEs exhibit several peculiar properties that are a direct consequence of the specific coupling mechanisms present in these materials. The behavior is especially interesting in the temperature region close to the nematic-paranematic phase transition, in which relatively small modifications of the external parameters such as stress, temperature or UV light intensity cause large modifications of the refractive index. This provides various possibilities to tune the grating properties and/or enhance the grating contrast according to the external stimuli.

In the LCE material used for our study the nematic-paranematic phase transition takes place at a relatively high temperature (*T*_c0_ = 82 °C). Consequently, the characteristic recording and relaxation times are relatively short, which means that time available for sample manipulation and/or different measurements, such as for instance angular dependence of the diffraction efficiency, is quite limited. To be able to investigate and control various phenomena contributing to the holographic recording in more detail, use of a similar LCE material with *T*_c0_ at lower temperatures or another type of LCE with long-lived azomesogenic groups would be convenient.
